# New perspectives on satisfaction and loyalty in festival tourism: The function of tangible and intangible attributes

**DOI:** 10.1371/journal.pone.0246562

**Published:** 2021-02-24

**Authors:** Jesús Molina-Gómez, Pere Mercadé-Melé, Fernando Almeida-García, Raquel Ruiz-Berrón

**Affiliations:** 1 Faculty of Economics Sciences, Department of Business and Management, University of Malaga, Malaga, Spain; 2 Faculty of Economics Sciences, Department of Statistics and Econometrics, University of Malaga, Malaga, Spain; 3 Faculty of Tourism, Institute of Tourism Intelligence and Innovation Research (i3t), University of Malaga, Malaga, Spain; Univerza v Mariboru, SLOVENIA

## Abstract

This research explains how attributes perceived during the festival celebration generate loyalty in terms of satisfaction. As regard, tangible aspects (festival entertainment and aesthetics) and intangible aspects (escapism and education) shall be differentiated. A theoretical model is proposed, which explains the effects of festival attributes on satisfaction and loyalty through structural equation modelling. The model was estimated with a sample of 440 people attending Weekend Beach Festival in Spain. The research proves the relationship between attributes and loyalty through satisfaction as a moderating variable; likewise, tangible attributes are deemed to have a greater influence on loyalty, specifically, the aesthetic/environment experience.

## Introduction

Nowadays, festivals are considered to be a fundamental pillar within the tourism industry. Festivals are valued, among other characteristics, for their ability to create an image in destinations and for being a tourist offering itself [1]. According to [2], this experiential product is essential for tourism promotion and is a clear marketing tool for the destination. Festivals play an increasingly more important role in tourist activity, thus relating them to various topics:

1. Academic interest on this topic is global, therefore studies can be found in any region around the world, whether in Europe [3], the Middle East [4], Asia [5], Africa [6], North America [7] and Oceania [8].
2. In recent years, research has focused on the analysis of visitors’ characteristics: the segmentation of attendees [2], their behaviour in theoretical models [9], attendees’ characteristics [10], satisfaction [5] and the loyalty created by festivals in their users [7].

Many authors define festival tourism as an experience product and a tourist offering made up of experiential aspects [7, 11]. On the other hand, festival tourism focuses on experience as one of the main competitive advantages, so much so that [12] defend the existence of an experience economy as a fundamental variable to value festival consumers’ behaviour. For these authors, festival consumers’ behaviour lies in the festival’s educational experience, entertainment, escapism and aesthetics. Organisers wish to create an environment for a satisfactory experience; they must generate an interest to return [13]. According to [14], the factors which contribute to creating satisfaction and loyalty in festival tourism are the festival’s activities, authenticity-uniqueness, sales concessions, environment, escape and socialisation throughout the event.

Some studies which analyse festival-goers’ loyalty highlight that festival’s characteristics influence loyalty through festival experience [3, 15]; other studies consider the type of attribute and perceptions on loyalty [14]. However, no focus has been made on the specific weight of each attribute and the festival experience to explain their effect on festival attendees’ satisfaction and loyalty.

**Fig 1:**
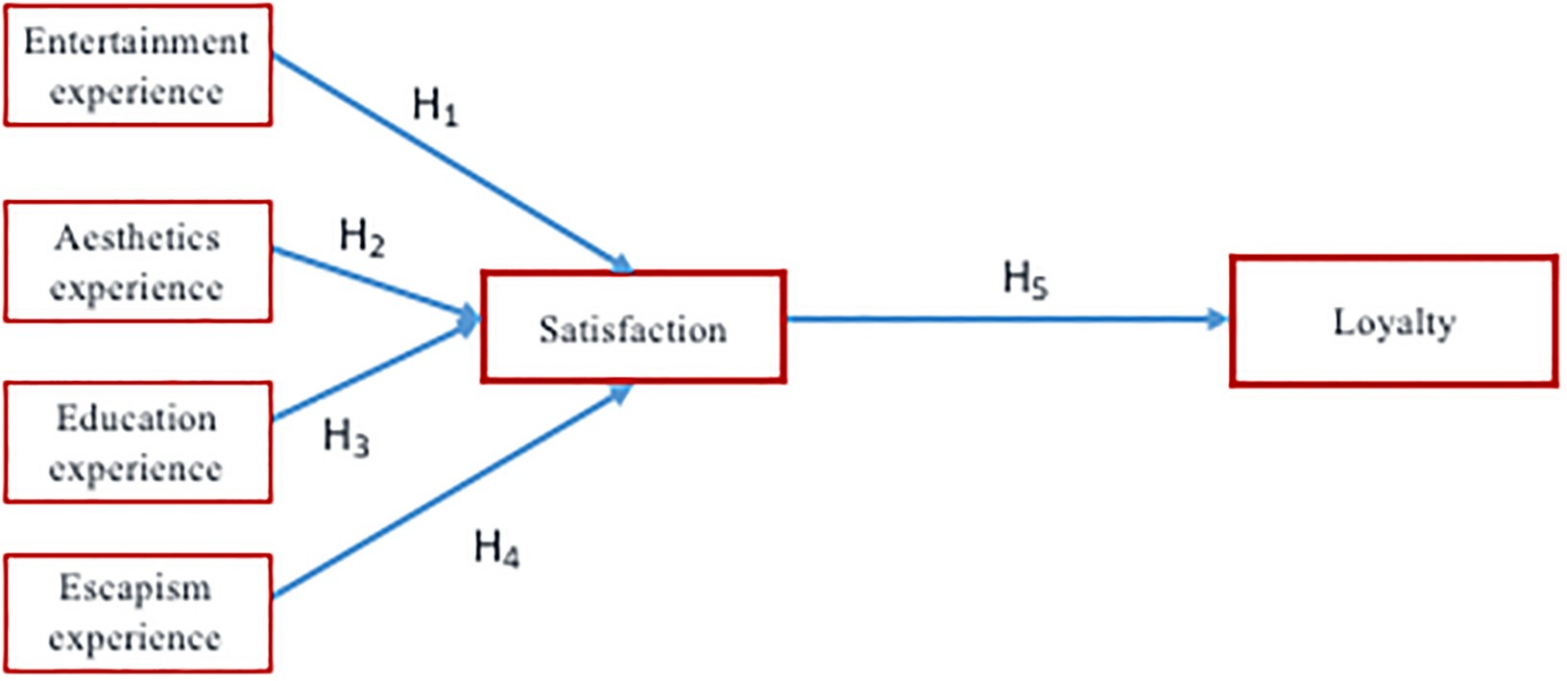
Research model proposal.

The aim of this study is to demonstrate the effect of the tangibility and intangibility of attributes on loyalty through satisfaction in festival tourism, bearing in mind the distinctive value of attributes (Fig 1). This research delves into previous studies which have analysed relationships between categories and loyalty and satisfaction in festivals [3, 9, 14, 15]. This study provides a new analysis on the distinctive role of festival attributes and experiences with regard to loyalty and satisfaction. This research represents an advance on the contributions of [12] in relation to the experiences and analyses of [14] on tangible and intangible attributes. Specifically, following the recommendation of [14]: *a future festival study could use structural equation modelling to test the hypothesis that tangible attributes influence loyalty through attendee satisfaction*, *whereas intangible attributes influence loyalty directly* (p.216). Thus, we apply structural equation modelling to find out the impact of tangible and intangible attributes on satisfaction and loyalty based on experience.

Likewise, this research is aimed at increasing knowledge on festival attendees’ perceptions and obtaining useful results for festival managers. We believe that a better analysis of the experiences and attributes of festivals clearly influence a more suitable management of these events. These two above-mentioned elements help to design the offer, distribution and sale of festivals, and allow a better understanding of the behaviour and evolution of the market, and explain the role of satisfaction and loyalty as essential elements in the competitiveness of festivals.

To this end, a structural equation model shall be used in order to discover the specific weight of each of the attributes of a festival and their relation with the variables of satisfaction and loyalty. The research is conducted on the basis of a survey on 440 attendees of the music festival Weekend Beach Festival in 2018 (WBF-2018), located on the Costa del Sol, in Andalusia (Spain).

## Literature review

### Attributes and experience in festival tourism

Consumers have experienced a process that pursues the satisfaction of psychological needs, such as inspiration, authenticity and sense of belonging to a community [16]. It is also important to remember that modern-day tourists seek to be informed and demand a more personal and memorable experiential offering. Thus, companies have been forced to reconsider their business model, since only organisations which are prepared to offer an adequate consumption experience shall succeed in the market [17].

#### Festival attributes

Many studies have been conducted on festivals, with several of them focusing on the aspects and attributes that influence on these events. [18] analysed the influence of four festival aspects (programme, souvenirs, food and facilities) on the value perceived and satisfaction; [14] carried out a meta-analysis in which they analysed 66 festival studies and identified the existence of six key attributes that attendees perceived at these kinds of events: programme, authenticity, concessions, environment, enjoyment and socialisation. These authors propose a classification according to the tangibility and intangibility of attributes, in which programme, authenticity, concessions and environment are tangible aspects and socialisation and enjoyment are intangible.

#### Experience in festival tourism

One of the first definitions of experience was described as “a subjective mental state felt by visitors during service delivery” [11, p.166]. [19] understands experience as the result of a group of reflections that originate during moments of conscience. [20] considers experience as a collection of relationships between the customer and a product/company to create a comfortable sensation.

[12, 21] proposed a consumer experience analysis framework which included four types of economic offerings: merchandise, goods, services and experiences; while the first three offerings are external, the experience solely exists in the mind of individuals; they separated service and experience from an economic perspective, creating the theory of the experience economy and identifying four areas of consumer experience: education, entertainment, escapism and aesthetics. The experiential link that a consumer may perceive depends on their participation and relationship with their environment, which result in a more intense and stronger experience [12]. Different studies have been developed on the basis of this theory, concluding that the dimensions of consumer experience are based on feeling, learning, and being and doing, respectively [22]. Lastly, [14] related the experience at festivals with the programme, authenticity, concessions, environment, enjoyment and socialisation.

Table 1 presents a list of all of the authors who have investigated these attributes (tangible and intangible) in connection with experience, satisfaction, loyalty and festival tourism.

**Table 1:** Authors and research on festival tourism.

Attributes		Authors
Tangible	1	[3, 7–9, 13, 15, 18, 23–43].
	2	[8, 10, 33, 36, 44–46].
	3	[3, 9, 26, 28–31, 34, 42, 47, 48].
	4	[3, 15, 18, 23, 30, 34, 38, 39, 41, 42, 45, 48].
	7	[3, 7–9, 13, 15, 23–27, 29, 33, 35, 38, 40–42, 49–55].
	8	[4, 6, 8, 33, 36, 46, 50, 55, 56].
	9	[3, 9, 26, 28, 29, 42, 48, 51, 52, 55].
	10	[3, 4, 7, 9, 15, 23, 25, 26, 29, 33, 38, 41, 42, 48, 51, 52, 54, 57, 58].
Intangible	5	[3, 8, 10, 13, 15, 24, 29, 46].
	6	[3, 8, 10, 13, 18, 24, 33, 38, 59].
	11	[3, 6, 8, 13, 15, 29, 46, 54, 56, 60, 61].
	12	[3, 8, 13, 33, 38, 50, 59].

1.Activities-Satisfaction	2.Authenticity -Satisfaction	3.Concessions-Satisfaction	4.Environment-Satisfaction	5.Enjoyment-Satisfaction	6.Socialisation-Satisfaction
7.Actividades-Loyalty	8.Authenticity -Loyalty	9.Concessions-Loyalty	10.Environment -Loyalty	11.Enjoyment—Loyalty	12.Socialisation- Loyalty

Source: Own elaboration.

Having presented the classification of festival studies according to the type of attribute and their connection with satisfaction and loyalty, studies relating to the hypothesis proposed in this research are analysed, linking experiences with tangible and intangible attributes and thus establishing a connection between these elements. Table 2 presents the order and correspondence between experiences [12] and related attributes [14] according to their tangibility and intangibility.

**Table 2:** Relationship between experiences and attributes according to type.

Type of Attribute	Experiences	Attributes
**Tangible**	Entertainment	Activity programme
	Aesthetics	Authenticity
		Concession
		Environment
**Intangible**	Education	Socialisation
	Escapism	Enjoyment

Source: Own elaboration.

In the following two sections experiences and attributes are related in accordance with Table 2.

### Tangible experiences and attributes

#### Entertainment and festival programme

One of the key aspects to understand the role of experience in the analysis of festivals is entertainment, which is a crucial aspect for satisfaction and loyalty. The entertainment experience in the festival context occurs when people passively watch other peoples’ performances [54]. The attribute linked to entertainment is usually found in the festival programme [14]. In Norway the quality of a jazz concert was measured based on the choice of artists, sound quality and programme [40]. [8] measured this attribute through activities such as wine, food and entertainment; [3] evaluated an Italian philosophy festival using the topics included in the programme. Two Korean festival programmes were evaluated through aspects such as enjoyment, diversity, amazement, correct organisation and planning [9, 29].

An example of entertainment analysis as an economic experience is the study conducted by [54]. This study was applied to a university festival in Iowa in which they identified that the different festival attributes affected the memory experienced and loyalty. Loyalty also demonstrates a connection with entertainment. [35] analysed a sporting event which confirmed the link of satisfaction and loyalty with entertainment and the competitive tournament. Bearing in mind the aforementioned studies, the following hypothesis is proposed.

H1 Entertainment will significantly predict satisfaction with the festival.

#### Aesthetics and festival environment

The aesthetic experience describes the evaluation of the physical aspects, mood or environment of the festival [21]. Over the years, studies have differentiated between *servicescape* and *festivalescape*. The first term is defined as the environment or influence on consumer’s feelings and behaviour [62]. In contrast, *festivalescape* refers to the general environment that attendees may perceive [30]. [63] consider the environment to be the essential motivation to attend the festival. [30] highlight the importance of *festivalescape* on satisfaction and loyalty. Subsequently, [22] verified the existing link between aesthetics and satisfaction perceived by attendees.

Regarding aesthetics and environment, [14] find authenticity to be a festival attribute: the authenticity of the local culture, environment and food, drinks and souvenir selling points. These authors understand the environment or atmosphere to be the festival’s location. Many studies have researched the attribute of festival environments and most of them highlight the importance of hygiene, safety, accessibility and resting areas [9, 15, 33].

Souvenir shops and food stalls have a relevant role at festivals, leading to various studies [14, 29]. At the Ginseng festival in Korea, [52] highlighted food and memory as significant variables. They also linked environment and accessories (souvenirs and food) with loyalty.

The aesthetic experience attribute is found in the authenticity and properties that are discovered in the town in which the festival is held [14]. At a cultural festival in Mongolia, research was conducted on the learning of the culture and the unique environment recreated [36]. [33] highlighted that floats, costumes, music and the city’s image are essential elements of the Patras carnival in Greece. At an Irani artisan festival, [4] highlighted the influence of the local staff, traditional exhibition and unique environment. Satisfaction in relation to the authenticity of festivals’ local characteristics has been approached by many authors [44–46]. Loyalty is also positively related to this attribute [6, 50, 55, 56]. On the basis of the studies analysed, the following hypothesis is proposed:

H2 The festival aesthetics will significantly predict satisfaction with the festival.

### Experiences and intangible attributes

#### Education and socialisation at festivals

The educational dimension is considered as the participants’ need to know, to experience new skills and abilities that may help intellectual and physical growth (Table 2) [12]. Educational development within experiences continues to grow [64], positioning self-education as a fundamental motivation for festival attendees [65]. [66] confirm this dimension at the Sidmouth festival, which was one of the reasons for their presence. The socialisation aspect is linked to educational experience, that is, social relationships made at the festival [14]. Relations with other people, the sense of belonging and proximity were crucial to evaluate social identity [3]. [24] also relates the intangible feeling of socialisation at these events with satisfaction and loyalty. At a charity golf event, [59] identifies familiarity as a sense of belonging and a group link. On the basis of the studies analysed, the following hypotheses is proposed:

H3 The educational experience will significantly predict satisfaction with the festival.

#### Escapism and enjoyment at the festival

Escapism is defined as the participants’ desire to get out of their routine where they are often stuck in, to escape reality, to live new experiences that change their routine (Table 2) [67]. In [24] analysed a blues music festival in a Turkish city where attendees commented that, for them, the festival was an escape and a novel experience. The representative attribute of escapism experience is enjoyment [14].

Attendees’ pleasure or hedonism was evaluated using phrases such as: the customer was satisfied; they appreciated the feeling of escapism and enjoyed the experience [3]. The following hypotheses is proposed for this research:

H4 The escapism experience will significantly predict satisfaction with the festival.

#### Satisfaction at festivals

According to (p.54) [68], satisfaction is defined as “*an evaluation based on the global purchase and consumption experience of a good or service in time*”; subsequent studies broadened knowledge on satisfaction, concluding that two precedents existed: the satisfaction of properties and information. Satisfaction of properties is explained as “*a subjective satisfaction judgement resulting from observations of the attribute performance*” (p.17) and the satisfaction of information as “*a subjective satisfaction criteria of the information used when choosing a product”* (p.18). Satisfaction has not been included as an attribute in previous studies, although it has been mentioned as a key word along with festivals [9] or visitors [43].

Regarding the experience, [69] researched festival context and highlighted that satisfaction would be explained as a general evaluation of the individual’s experience at a festival. In recent years, a positive relationship is observed between satisfaction and loyalty and it is concluded that satisfaction determines attitude and the willingness to consume is a result of the experience perceived by the customer with a product or service [70]. The more the customers are satisfied with the product or service, the more they shall be willing to recommend it [71].

### Loyalty at festivals and its connection with satisfaction

[70] *defined loyalty as a strong commitment to buy back or endorse a consistent product or service again in the future*, *leading to repeat purchases of the same brand*, *despite influences and marketing efforts that can potentially cause a disruption and behaviour change* (p. 34). According to this author, loyalty is a multidimensional concept that develops in several stages of loyalty: affective loyalty, cognitive loyalty, action loyalty and conative loyalty [70]. Loyalty can be explained from variables such as attitude or behaviour [72]. Usually, in marketing studies, loyalty has been analysed as a single construct that incorporates the aforementioned variables [73, 74]. In this study, the measurement of loyalty was defined by adapting the [75] scale, related to the theory of planned behaviour (TPB) and the tendency to repeat.

Other authors consider loyalty in festival tourism as the predisposition of users to repeat certain behaviour [15], which implies the intention to attend festivals again [26] and recommend them to others [49]. Said repetition has been analysed in the tourism field with regards to loyalty regarding specific destinations. The authors [76] adapted Best’s loyalty index to a tourist destination (Seville, Spain) taking into account the number of repeated visits to the destination [77]. According to these authors, high loyalty implies more than three visits to the same destination. However, it is not very common to study loyalty at festivals through this type of specific measures. The number of times a festival is attended is not analysed so much as the aspects that affect loyalty, the effects of loyalty and the recommendation to attend a festival.

Some studies measure loyalty as future expenses; for example, purchasing wine after attending an Australian wine festival [78] or the intention to commit to donating in order to help a cause after attending a charity festival [59]. [33] states that attendees’ intentions are directly influenced by the festival experience. Likewise, several studies that have linked satisfaction and loyalty, observed this fact in catering services [79, 80].

In other research conducted on festivals, satisfaction is a positive precedent towards loyalty [23, 81]; more recently, a study analysed the effect caused by the four attributes (programme, souvenirs, food and facilities) on the value perceived and, consequently, on satisfaction and loyalty. This study concluded that satisfaction and loyalty may be improved through the value perceived, since they are positively related [18]. Other studies show how emotional solidarity is directly influenced by tourist loyalty and satisfaction. A good example is the study carried out on the Cape Verde Islands, where it is shown the existence of a positive relationship between the variables [82]. Likewise, the case of the study conducted in Karkala (India) at a religious festival generates an emotional solidarity that has a positive effect on loyalty and, partially, on satisfaction [83].

H5 The festival satisfaction will significantly predict loyalty with the festival.

## Methodology

### The study setting: Festival description

The study was carried out on the “Weekend Beach Festival (WBF)” held on the Costa del Sol, Torre del Mar, Spain. The festival was first held in 2014 and was one of the most cutting-edge tourism proposals on the coast of Malaga. The festival had a 67,000 m^2^ enclosure with three stages just metres from the beach, hosting current national and international artists. The festival was held for four days in July and reached a record figure of 140,000 attendees in 2008; the growth experienced over recent years has been a breathtaking 42% in comparison to 2015. It is currently number 11 in the official Spanish festival ranking according to the Social Network Festival Awards 2018. The tourists’ staying in the town has increased each year until it reached 100% capacity during its celebration over recent years. 20% of attendees are locals and 80% of them come from the rest of Spain and abroad.

### Research instrument

Information gathering was carried out using a structured questionnaire. It was handed out personally in bars, establishments and streets in the town of Torre del Mar, Malaga, the city in which the festival was held. A two-stage method was used to choose the sample. In the first step, the method for selecting the sample was proportional and stratified according to the attendees’ origin. In the second stage, the respondents were selected in a non-probabilistic way for convenience. This method is suitable when it is difficult to find the participants and it is equally valid [84]. Prior to the fieldwork, a group dynamic was used to explain the procedure of gathering information. Furthermore, a pre-test was taken by 20 people to evaluate their understanding of the questions.

Fieldwork was carried out from June to July 2018, achieving 440 valid questionnaires, with a sampling error of 4.7% and a confidence level of 95%. The response rate was 89%. The research we have carried out has been based on anonymous surveys that respect ethical and analytical standards in the field of social sciences and do not require the prior approval of an ethical committee, in accordance with national and European legislation.

The questionnaire does not ask about sensitive social aspects such as race, religion, sexual orientation, diseases, etc. The items are focused on the analysis of the analysis of the analysed phenomenon (festival). The funding source has not requested the analysis of any particular group. All authors respect the rules of confidentiality and ethics regarding the analysis of data and results, in accordance with national, European standards and international agreements.

In order to have a greater representation of data, a multi-stage sampling by quotas was carried out based on the gender (Table 3). With regards to the respondents’ profile, 50.4% of the participants were female and 49.6% were male. Most respondents were between 20 and 24 years of age (51.8%), followed by those between 15 and 19 (23.6%).

**Table 3:** Technical data of the sample.

**Data collection period**	15th June 2018–15th July 2018
**Population**	140,000 attendees
**Type of sample**	Geographic quota sampling and gender
**Size of sample**	440 respondents
**Type of survey**	Face-to-face questionnaire
**Sample error**	4.7%
**Confidence level**	95%

Source: Own elaboration.

### Measurement scale of variables

The nature of the causal links is reflective [85, 86], because they are more suitable for defining attitudinal features [87, 88]. The constructs analysed on economical experiences (entertainment, aesthetics, and education) were measured against 4 items each one, and 3 for escapism construct, through an adaptation of the scale proposed by [9, 54]. Satisfaction was measured against 3 items based on the consumer’s literature and their behaviour [74, 89]. Loyalty is measured against 3 items through an adaptation of the scale proposed by [75]. The total number of items is 21. The evaluation of the constructs was estimated on a Likert scale of seven points that ranged from (1) strongly disagree to (7) strongly agree. The scale with the items used can be seen in Table 4 and the annex of Supporting information.

**Table 4:** Confirmatory factor analysis. Psychometric properties.

Construct and scale items		Mean	SD	Standardised loadings	Average loads	α	AVE	CRI
**Entertainment Experience**								
ENT 1	The WBF concerts were fun to watch	6.277	0.879	0.788	0.812	0.9353	0.66	0.89
ENT 2	Seeing the WBF concerts captivated me	5.668	1.126	0.763				
ENT 3	I enjoyed watching the concerts at WBF	6.464	0.759	0.850				
ENT 4	It was really entertaining to watch the concerts at WBF	6.373	0.851	0.848				
**Aesthetics Experience**								
EST 1	I felt a real sense of harmony at WBF	5.259	1.382	0.702	0.780	0.9205	0.61	0.86
EST 2	For me, the WBF setting was pleasant	5.864	0.991	0.817				
EST 3	Being at WBF was really pleasant	6.191	0.904	0.818				
EST 4	The WBF setting was very attractive	5.723	1.214	0.783				
**Education Experience**								
EDU 1	My experience at WBF has been useful to increase my knowledge	4.745	1.414	0.845	0.755	0.753	0.58	0.84
EDU 2	I learned a lot from my experience at WBF	4.959	1.360	0.823				
EDU 3	Attending WBF heightened my curiosity to learn new styles	5.573	1.449	0.605				
EDU 4	I classify my experience at Weekend Beach Festival as highly educational	4.073	1.577	0.746				
**Escapism Experience**								
ESC 1	At WBF I felt like I was living in a different place or time	5.550	1.392	0.864	0.795	0.8801	0.64	0.84
ESC 2	The WBF experience allowed me to imagine I was someone else	4.023	2.017	0.780				
ESC 3	I was able to completely escape reality at WBF	5.223	1.660	0.742				
**Satisfaction**								
SAT 1	Overall, I am satisfied with WBF	5.350	1.401	0.831	0.802	0.918	0.65	0.85
SAT 2	As a whole, I am happy with the WBF	6.064	1.337	0.674				
SAT 3	I believe attending the WBF was the right decision	5.841	1.163	0.902				
**Loyalty**								
LOY 1	I will spread positive word-of-mouth about WBF	6.414	1.143	0.881	0.909	0.918	0.83	0.93
LOY 2	I will continue to attend WBF	6.445	1.117	0.927				
LOY 3	I will recommend WBF to my friends	6.564	0.920	0.918				

Source: Own elaboration.

Statistical analysis of the data is based on a structural equation model (SEM) to identify the specific weight of each of the attributes and their relationship with the two variables: satisfaction and loyalty. For the analysis of the model being studied, the recommendations made by [90] were followed. Additionally, STATA.15 software was used for their estimation. At the beginning, the goodness of fit of the measurement model was analysed on the basis of the confirmatory factor analysis (CFA) to evaluate the psychometric properties. Then, the causal relationships have been analysed on the basis of the structural equation modelling (SEM).

## Results

### Analysis of the psychometric properties of the measurement model

In the following tables, the psychometric properties are evaluated using the main measures of reliability, validity and goodness and the matrix of correlations between factors.

First, in order to assess common method bias, we applied Harman’s single factor method [91–93]. The first factor that has been obtained by applying the exploratory factor analysis to all the variables of the model represents 29.92% of the total variance, being below the threshold of 50%.

In Table 4 it is observed that the variables of the theoretical model fulfil all of the measures of reliability. The average variance extracted (AVE) is higher than 0.5 [94], the Cronbach alpha (α) is higher than 0.7 [95] and the composite reliability index (CRI) is higher than 0.7 [94]. The measures of validity are also adequate; the coefficients of standardised loadings are higher than 0.5 and its averages are higher than 0.7 [96]; therefore, convergent validity is confirmed. This means that the items of a construct are co-related with each other [96].

The amount of variance that each construct captures from its indicators (AVE) is higher than the variance that said construct shares with other constructs in the model; therefore, the discriminating validity of the measurement model is confirmed [94]. Table 5 provides the square root of the AVE of each construct, which is higher than the correlations with other constructs in the model. With regard to the measures of goodness, the root mean square error of approximation (RMSEA) is appropriate, since it is lower or equal to 0.08 [97] and the Tucker-Lewis index (0.91) and the comparative fit index (0.93) are close to 1 [98]. The only indicator that does not comply is the χ^2^ (480.29). However, this is not considered to be a limitation, since it is very sensitive to the sample size [93] and, frequently, the hypothesis of a good fit of the model is rejected in large samples even if it is significant [99].

**Table 5:** Test of discriminant validity. Correlations among the constructs.

**Factor**	**Entertainment**	**Aesthetics**	**Education**	**Escapism**	**Satisfaction**	**Loyalty**
**Entertainment**	0.813					
**Aesthetics**	0.585	0.781				
**Education**	0.319	0.451	0.760			
**Escapism**	0.321	0.483	0.400	0.797		
**Satisfaction**	0.403	0.495	0.296	0.371	0.808	
**Loyalty**	0.330	0.169	0.162	0.057	0.197	0.909

Note: Diagonal indicates the square root of AVE. Correlations are reported in the lower half of the matrix.

Source: Own elaboration.

### Analysis of the structural relations and hypotheses proposed

Table 6 analyses the standardised coefficients of the structural relationships of the theoretical model proposed. The ordinal data was analysed, taking in account non-normality problems. The model was estimated using maximum likelihood with Satorra-Bentler adjustments [100, 101]. In this case, the statistical indices are robust.

**Table 6:** Evaluation of structural models.

Hypothesis	Structural relationship	Coefficient	Value t*	Contrast
H_1_	Entertainment → Satisfaction	0.2816	4.28***	Supported
H_2_	Aesthetics→ Satisfaction	0.3675	5.98***	Supported
H_3_	Education → Satisfaction	0.1086	1.86*	Supported
H_4_	Escapism → Satisfaction	0.1607	2.28**	Supported
H_5_	Satisfaction → Loyalty	0.1832	2.75***	Supported
Goodness of fit				
S-Bχ2		CFI	TLI	RMSEA
480.29 (p = 0.000)		0.93	0.91	0.08

Note:

*: p-value<0.1;

**: p-value<0.05;

***: p-value<0.01.

Source: Own elaboration.

**Fig 2:**
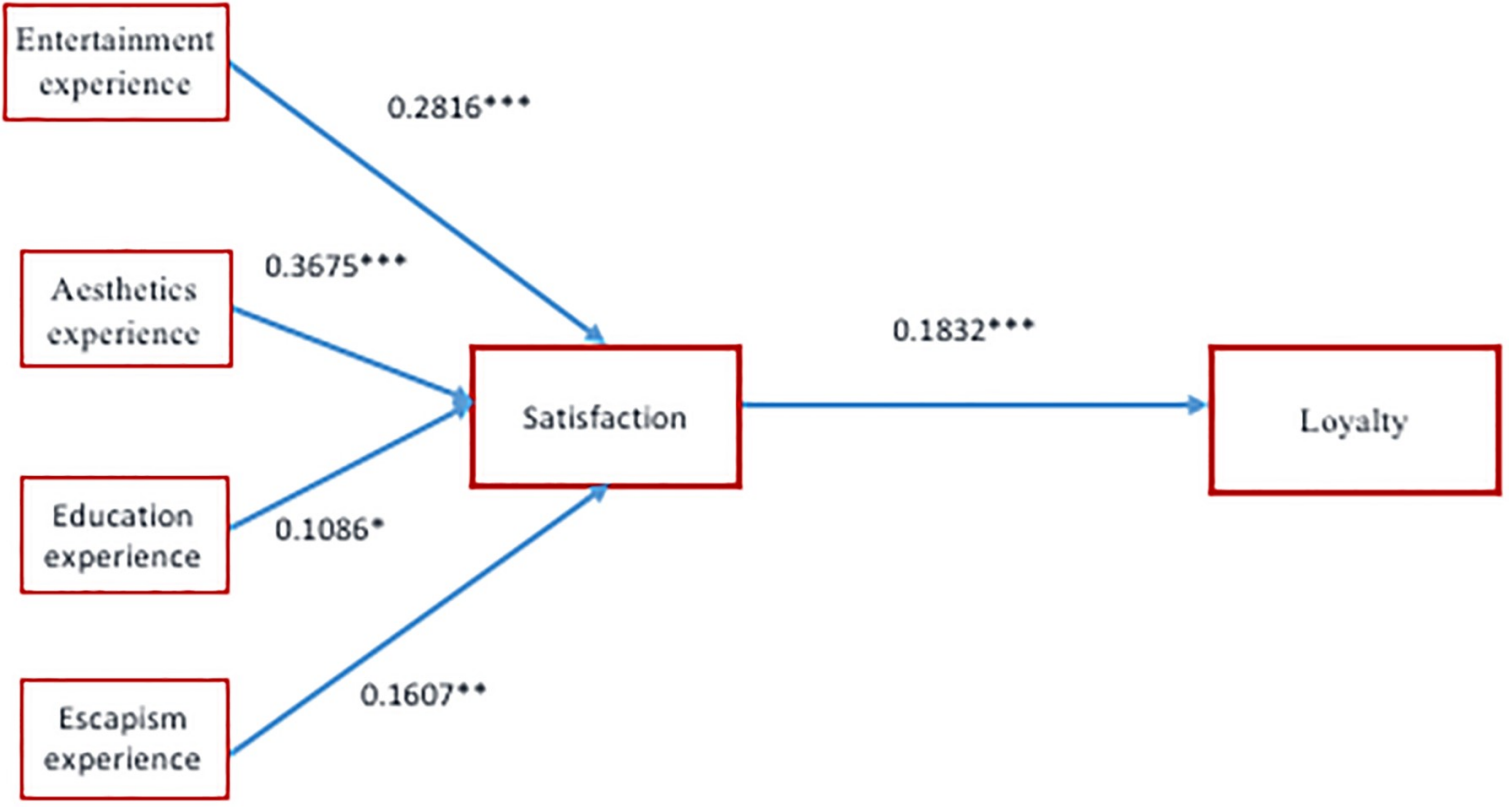
Structural model results.

It can be observed that all of the causal relationships are statistically significant (H1 and H5 are supported). In this sense, it can be stated that a positive causal relationship exists between entertainment experience and satisfaction (β = 0.2816), aesthetics experience and satisfaction (β = 0.3675), education experience and satisfaction (β = 0.1086), escapism experience and satisfaction (β = 0.1607) and, finally, between satisfaction and loyalty (β = 0.1832). Therefore, the results show a direct relationship between the different experiences and satisfaction, as well as satisfaction and loyalty. The most intense of the causal relationships between perceived destination attributes and satisfaction occurs between the aesthetics experience and satisfaction, followed by the entertainment experience, escapism and, finally, the education experience. This SEM analysis provides empirical evidence that tangible attributes, such as entertainment and aesthetics, have a greater influence on satisfaction and loyalty than intangible attributes (education and escapism). To analyse the predictive capacity of the model, the coefficient of determination (R^2^) has been calculated, and the results obtained are 0.39 for satisfaction and 0.14 for loyalty. Fig 2 shows the model with its respective structural coefficients.

## Discussion, conclusions and implications for management

The results confirmed the impact of the different experiences on satisfaction and loyalty in the festival’s context; the results gathered, thanks to the analysis of the structural equation model, as recommended by [14], shows how tangible and intangible attributes generate satisfaction and loyalty in attendees from the experience, providing tangible attributes (programme, authenticity, concessions and atmosphere) a higher level of satisfaction. In contrast, in the study conducted by [54], on the basis of a regression analysis, a direct positive relationship is found between entertainment and aesthetics experience, and loyalty; however, this same relationship is not evidenced between education and escapism experience and loyalty; that is to say, only a relationship between tangible attributes and loyalty is found. By contrast, our research points out the existence of a relationship between tangible and intangible attributes and loyalty through satisfaction, having tangible attributes a higher impact on loyalty than the intangible ones.

The conclusions provided by this study may be used by festival organisers to discover which specific experiences and attributes generate the greater satisfaction and loyalty in attendees. This research highlights that tangible attributes are linked to entertainment and aesthetics experiences, being entertainment a key element at festivals for attendees with regards to satisfaction and loyalty.

Knowing festival audience is essential in order to identify what type of entertainment to offer. Thus, a highly-educated audience shall show more musical interest for classical music or traditional theatre [102]. In contrast, a middle-class audience shall be more interested in music such as pop, hip-hop, jazz or modern dance [67].

Adequate ground facilities, food quality, souvenirs and authenticity of the location in which the festival is held impact on the aesthetic experience. Thus, variables are deemed crucial for festival-goers and, as a result, they must be thoroughly looked after. An example of the foregoing is the Greek carnival in Patras, where the quality of the event depended on the quality of the food, drinks and hygiene of the toilets [33]. In the case of the Punggi Ginseng festival (Korea), food and memory were essential variables of the event [52]. The authenticity of the artisan Turkmen festival in Iran was based on unique products, local staff, traditional presentation and unique atmosphere [4]. Thus, festival organisers are recommended to make the most of local characteristics to boost the festival’s authenticity [67].

With regards to festivals’ intangible attributes, socialisation and enjoyment are related to the education and escapism experiences respectively. The aforementioned attributes generate a positive effect on satisfaction and loyalty, although to a lesser extent than tangible attributes. Intangible attributes and experiences are fundamental for planning festivals, once analysed the study conducted by [3] regarding socialisation and learning, in which attitudes such as the sense of belonging, concordance and identity with other customers and proximity were identified. In this sense, [59] identified the sense of belonging at a charity golf event and [67] suggested the inclusion of personal growth as a variable that increases attendance to these events.

Regarding enjoyment and escapism experience, achieving the enjoyment of festival attendees is a way of accomplishing a feeling of escapism. This topic has been discussed by various authors, some of them highlighting escapism or novelties [24]; other studies highlight the enjoyment of the experience [3] and fun [13].

Therefore, this study may be concluded with evident results. On the one hand, it has been demonstrated that the satisfaction of festival tourists has a direct impact on loyalty and that tangible attributes generate a greater influence; particularly attributes which relate to the aesthetics experience, rather than those which relate to the entertainment experience (Table 6) (Fig 2); therefore, the first, second and fifth hypotheses from our research are accepted. On the other hand, to a lesser extent, intangible attributes generate a positive effect on satisfaction and loyalty, having escapism experience attributes a greater influence than education experience attributes (Table 6) (Fig 2); thus, the third, fourth and fifth hypotheses from our research are accepted. Therefore, tangible attributes present a greater causal relationship than intangible attributes in satisfaction and loyalty, according to the analysis model analysed in this research.

The practical implications of this research highlight that event managers should prioritise entertainment experience and aesthetics experience, because they are the attributes that have the highest influence on the satisfaction and loyalty of festival goers. Moreover, these managers should not forget tangible aspects such as education experience and escapism experience, because although they influence to a lesser extent, they also present a remarkable causal relationship with satisfaction and loyalty. This is in line with other authors who have studied the relationship and satisfaction through attributes [73, 74]. The experience of a successful activity is crucial to the long-term competitiveness of destinations [103].

### Limitations and future research

When conducting this research, we came across some limitations which are discussed as follows. The festival participants studied are attendees to a current music festival (Weekend Beach Festival, WBF-2018) and, consequently, the data obtained may not be applicable to other festivals with different characteristics. With regards to creating the analysis scale, the results of the study indicate that tangible and intangible attributes only account for experiences. It would be appropriate for future studies to investigate other aspects such as emotions and experienced memories, which may be important for the analysis of affective variables. Another limitation of this study is the lack of attention paid to the interaction that visitors showed at the festival, emotional solidarity playing an important role in festival tourism, as described by the authors [104, 105].

Likewise, no control effect has been included in the model for this study. Future research should include some control variables such as service quality, perceived value and any other variable that is considered crucial to explain the model. Lastly, research on festivals could focus on other aspects of interest such as the change in perception of festivals by new generations. In future research, emotional solidarity in festival tourism will be taken into account as a key element to better explain the behaviour of visitors and their interaction with the destination [82, 105].

## Supporting information

S1 File (DOCX)

S2 File (DOCX)
