# Supplementary material for: New perspectives on satisfaction and loyalty in festival tourism: The function of tangible and intangible attributes
Source: PLoS One. 2021 Feb 24;16(2):e0246562. doi: 10.1371/journal.pone.0246562 (PMC7904192; doi:10.1371/journal.pone.0246562)
Supplement: S1 File — (DOCX) [file pone.0246562.s001.docx]

**Supporting information**

**Items and Scales of the questionnaire**

|  |  |  |
| --- | --- | --- |
| **Code** | **Item** | **Scale items** |
|  |  |  |
| **Entertainment Experience** | |  |
| ENT 1 | The WBF concerts were fun to watch | 7-point Likert-type scale |
| ENT 2 | Seeing the WBF concerts captivated me | 7-point Likert-type scale |
| ENT 3 | I enjoyed watching the concerts at WBF | 7-point Likert-type scale |
| ENT 4 | It was really entertaining to watch the concerts at WBF | 7-point Likert-type scale |
| **Aesthetics Experience** | |  |
| EST 1 | I felt a real sense of harmony at WBF | 7-point Likert-type scale |
| EST 2 | For me, the WBF setting was pleasant | 7-point Likert-type scale |
| EST 3 | Being at WBF was really pleasant | 7-point Likert-type scale |
| EST 4 | The WBF setting was very attractive | 7-point Likert-type scale |
| **Education Experience** | |  |
| EDU 1 | My experience at WBF has been useful to increase my knowledge | 7-point Likert-type scale |
| EDU 2 | I learned a lot from my experience at WBF | 7-point Likert-type scale |
| EDU 3 | Attending WBF heightened my curiosity to learn new styles | 7-point Likert-type scale |
| EDU 4 | I classify my experience at Weekend Beach Festival as highly educational | 7-point Likert-type scale |
| **Escapism Experience** | |  |
| ESC 1 | At WBF I felt like I was living in a different place or time | 7-point Likert-type scale |
| ESC 2 | The WBF experience allowed me to imagine I was someone else | 7-point Likert-type scale |
| ESC 3 | I was able to completely escape reality at WBF | 7-point Likert-type scale |
| **Satisfaction** | |  |
| SAT 1 | Overall, I am satisfied with WBF | 7-point Likert-type scale |
| SAT 2 | As a whole, I am happy with the WBF | 7-point Likert-type scale |
| SAT 3 | I believe attending the WBF was the right decision | 7-point Likert-type scale |
| **Loyalty** |  |  |
| LOY 1 | I will spread positive word-of-mouth about WBF | 7-point Likert-type scale |
| LOY 2 | I will continue to attend WBF | 7-point Likert-type scale |
| LOY 3 | I will recommend WBF to my friends | 7-point Likert-type scale |
|  | | |
