# Supplementary material for: New perspectives on satisfaction and loyalty in festival tourism: The function of tangible and intangible attributes
Source: PLoS One. 2021 Feb 24;16(2):e0246562. doi: 10.1371/journal.pone.0246562 (PMC7904192; doi:10.1371/journal.pone.0246562)
Supplement: S2 File — (DOCX) [file pone.0246562.s002.docx]

**STATA OUTPUT**

. sem (entertainment -> P61, ) (entertainment -> P62, ) (entertainment -> P63, ) (entertainment -> P64, ) (entertainment -> satisfaction, ) (education -> P51, ) (education -> P

> 52, ) (education -> P53, ) (education -> P54, ) (education -> satisfaction, ) (escapism -> P91, ) (escapism -> P92, ) (escapism -> P93, ) (escapism -> satisfaction, ) (aesthe

> tics -> P101, ) (aesthetics -> P102, ) (aesthetics -> P103, ) (aesthetics -> P104, ) (aesthetics -> satisfaction, ) (loyalty -> P116, ) (loyalty -> P114, ) (loyalty -> P115,

> ) (satisfaction -> loyalty, ) (satisfaction -> P111, ) (satisfaction -> P112, ) (satisfaction -> P113, ), covstruct(_lexogenous, diagonal) vce(sbentler) standardized latent(e

> ntertainment education escapism aesthetics loyalty satisfaction ) nocapslatent

Endogenous variables

Measurement: P61 P62 P63 P64 P51 P52 P53 P54 P91 P92 P93 P101 P102 P103 P104 P116 P114 P115 P111 P112 P113

Latent: satisfaction loyalty

Exogenous variables

Latent: entertainment education escapism aesthetics

------------------------------------------------------------------------------------

| Satorra-Bentler

Standardized | Coef. Std. Err. z P>|z| [95% Conf. Interval]

-------------------+----------------------------------------------------------------

Structural |

satisfaction |

entertainment | .2816235 .0657576 4.28 0.000 .152741 .4105059

education | .1086811 .0584616 1.86 0.063 -.0059016 .2232637

escapism | .1607441 .0703995 2.28 0.022 .0227637 .2987245

aesthetics | .3675486 .061438 5.98 0.000 .2471323 .487965

-----------------+----------------------------------------------------------------

loyalty |

satisfaction | .1832041 .0666404 2.75 0.006 .0525914 .3138169

-------------------+----------------------------------------------------------------

Measurement |

P61 |

entertainment | .7013658 .0430284 16.30 0.000 .6170317 .7856999

_cons | 7.139168 .3575552 19.97 0.000 6.438373 7.839963

-----------------+----------------------------------------------------------------

P62 |

entertainment | .597721 .0462942 12.91 0.000 .5069861 .688456

_cons | 5.035313 .1810082 27.82 0.000 4.680543 5.390082

-----------------+----------------------------------------------------------------

P63 |

entertainment | .8354498 .0267445 31.24 0.000 .7830315 .887868

_cons | 8.516955 .4041186 21.08 0.000 7.724897 9.309013

-----------------+----------------------------------------------------------------

P64 |

entertainment | .8210454 .0294882 27.84 0.000 .7632495 .8788413

_cons | 7.485882 .3792154 19.74 0.000 6.742634 8.229131

-----------------+----------------------------------------------------------------

P51 |

education | .8848199 .0327268 27.04 0.000 .8206764 .9489633

_cons | 3.356514 .1804578 18.60 0.000 3.002823 3.710205

-----------------+----------------------------------------------------------------

P52 |

education | .709494 .0573754 12.37 0.000 .5970403 .8219477

_cons | 3.647646 .1641123 22.23 0.000 3.325992 3.969301

-----------------+----------------------------------------------------------------

P53 |

education | .4052919 .0580657 6.98 0.000 .2914852 .5190985

_cons | 3.846228 .2552991 15.07 0.000 3.345851 4.346605

-----------------+----------------------------------------------------------------

P54 |

education | .6498633 .0442159 14.70 0.000 .5632017 .736525

_cons | 2.583259 .1269502 20.35 0.000 2.334441 2.832077

-----------------+----------------------------------------------------------------

P91 |

escapism | .5698961 .0487368 11.69 0.000 .4743737 .6654186

_cons | 3.986303 .2664567 14.96 0.000 3.464057 4.508548

-----------------+----------------------------------------------------------------

P92 |

escapism | .8251673 .0531266 15.53 0.000 .721041 .9292936

_cons | 1.994564 .1047259 19.05 0.000 1.789305 2.199823

-----------------+----------------------------------------------------------------

P93 |

escapism | .6957171 .0547827 12.70 0.000 .5883451 .8030892

_cons | 3.146599 .1564666 20.11 0.000 2.83993 3.453268

-----------------+----------------------------------------------------------------

P101 |

aesthetics | .5011984 .0504729 9.93 0.000 .4022733 .6001234

_cons | 3.805199 .1852691 20.54 0.000 3.442079 4.16832

-----------------+----------------------------------------------------------------

P102 |

aesthetics | .7757088 .0333404 23.27 0.000 .7103629 .8410547

_cons | 5.918927 .202432 29.24 0.000 5.522167 6.315686

-----------------+----------------------------------------------------------------

P103 |

aesthetics | .8081323 .0378885 21.33 0.000 .7338722 .8823924

_cons | 6.844654 .326446 20.97 0.000 6.204832 7.484477

-----------------+----------------------------------------------------------------

P104 |

aesthetics | .702826 .0418094 16.81 0.000 .620881 .784771

_cons | 4.715026 .2546329 18.52 0.000 4.215954 5.214097

-----------------+----------------------------------------------------------------

P116 |

loyalty | .756363 .0509565 14.84 0.000 .6564901 .8562359

_cons | 7.149452 .5386709 13.27 0.000 6.093677 8.205228

-----------------+----------------------------------------------------------------

P114 |

loyalty | .8773396 .0350525 25.03 0.000 .8086379 .9460413

_cons | 5.625742 .4545196 12.38 0.000 4.7349 6.516585

-----------------+----------------------------------------------------------------

P115 |

loyalty | .9746437 .029717 32.80 0.000 .9163994 1.032888

_cons | 5.789538 .4605573 12.57 0.000 4.886863 6.692214

-----------------+----------------------------------------------------------------

P111 |

satisfaction | .6455219 .0554472 11.64 0.000 .5368474 .7541964

_cons | 3.967749 .219427 18.08 0.000 3.53768 4.397818

-----------------+----------------------------------------------------------------

P112 |

satisfaction | .4934999 .0692237 7.13 0.000 .3578239 .6291759

_cons | 4.641212 .3994022 11.62 0.000 3.858398 5.424026

-----------------+----------------------------------------------------------------

P113 |

satisfaction | .8703957 .054106 16.09 0.000 .7643498 .9764415

_cons | 5.377048 .2943556 18.27 0.000 4.800121 5.953974

-------------------+----------------------------------------------------------------
